# Interventions to improve participation in health‐care decisions in non‐Western countries: A systematic review and narrative synthesis

**DOI:** 10.1111/hex.12933

**Published:** 2019-06-09

**Authors:** Hankiz Dolan, Mu Li, Lyndal Trevena

**Affiliations:** ^1^ Sydney School of Public Health The University of Sydney Sydney New South Wales Australia; ^2^ Ask, Share, Know: Rapid Evidence for General Practice Decision (ASK-GP), Centre for Research Excellence The University of Sydney Sydney New South Wales Australia

**Keywords:** decision making, health communication, patient participation, patient‐centred care, systematic reviews

## Abstract

**Background:**

Patients' participation in medical decision making is an important aspect of patient‐centred care. However, there is often uncertainty about its applicability and feasibility in non‐Western countries.

**Objective:**

To provide an overview and assessment of interventions that aimed to improve patients' participation in decision making in non‐Western countries.

**Method:**

Ovid Medline, Embase, Cochrane Central Register of Controlled Trials, CINAHL, Ovid MEDLINE(R) Epub Ahead of Print, In‐Process, Other Non‐Indexed Citations, without Revisions and Daily Update and Database of Abstracts of Reviews of Effects, were searched from respective inception to February 2018. Studies were included if they (a) were randomized controlled trials, before‐and‐after studies and interrupted time series studies; (b) were conducted in non‐Western countries; (c) aimed to improve patients' participation in dyadic decision making; and (d) reported outcomes relevant to patient participation in decision making. Studies were excluded if they included children, were about triadic decision making or solely focused on information provision without reporting outcomes related to patient participation. Narrative synthesis method was used for data analysis and presentation.

**Results:**

A total of 17 studies, 6 RCTs and 11 non‐RCTs, were included across ten countries. Intervention strategies included patient and/or provider communication skills training, decision aids and a question prompt material. Whilst most of the studies reported increased patient participation, those interventions which had provider or patient training in communication skills were found to be more effective.

**Conclusion:**

Interventions to improve patient participation, within the context of dyadic decision making, in non‐Western countries can be feasible and effective if communication skills training is provided for health‐care providers and/or patients.

## 1 INTRODUCTION

Participation in decision making is a process where engaged patients and health‐care providers partake in shared decision making through the meaningful exchange of information and experiences.^1^ It is a key characteristic of patient‐centred health care, a paradigm that has become popular in recent decades, replacing more paternalistic health‐care models. Recent evidence shows that greater participation in health‐care decisions increases patients' satisfaction, improves patient‐provider relationships, facilitates medication adherence and decreases health‐care costs.^2^, ^3^ There is also emerging evidence that participation in decisions may reduce health inequalities experienced by vulnerable groups such as racial and ethnic minorities, low literacy groups and seniors.^4^ However, issues such as time constraints, patient characteristics, low health literacy and cultural factors are often reported as barriers to participative decision making, with some saying that it is impractical amongst certain groups.^5^, ^6^, ^7^

Globally, this paradigm shift was reflected in the pronouncement of the Alma‐Ata Declaration in 1979, a landmark moment calling for greater participation from individuals and communities in their health‐care planning and implementation.^8^, ^9^ More recently, the World Health Organization (WHO) published the Framework on Integrated People‐Centred Health Services (IPCHS), promoting Universal Health Coverage through equal, responsive, affordable and quality health‐care services.^10^ An important strategy proposed by this Framework is to engage and empower individuals and families.^11^ A number of strategies, including shared clinical decision making, were proposed. However, the report fell short of providing recommended strategies, concept analyses and best practices.

In many Western countries, policies have been developed to support patients' participation in health‐care decision making and the use of decision aids, question prompt lists and training for both clinicians and patients.^12^, ^13^, ^55^ For example, in Australia, the statement ‘I have a right to be included in decisions and choices about my care’ is part of the Australian Charter of Healthcare Rights.^14^ However, less is known about how to effectively involve patients in health‐care decisions in non‐Western countries. In these settings, some have argued that the concepts of ‘patient centeredness’ and ‘active participation’ are based on the Western ideology of individual autonomy and are therefore less applicable.^16^, ^17^ In cultures where individuals see themselves as agents of a family, community or a tribe, within a hierarchical community, health‐care professionals are often to be respected.^18^, ^19^, ^20^ Questioning by patients is to be avoided to bring harmony during encounters.^18^, ^19^, ^20^ Other factors that may be prevalent in some non‐Western countries are high patient loads, lack of skills in participatory communication amongst health providers, a lack of relevant research evidence and low health literacy amongst patients.^20^, ^21^, ^22^ These lead to the question of whether patient‐centred care, and more specifically, patients' participation in health decisions, is a feasible and appropriate strategy in non‐Western country contexts. This systematic review aims to identify interventions designed to improve adult patients' participation in health‐care decisions in non‐Western countries, assess their feasibility and synthesize factors that influence their effectiveness.

## 2 METHODS

This systematic review is reported in accordance with preferred reporting items for systematic reviews and meta‐analyses (PRISMA) (Appendix S1).^23^

### 2.1 Inclusion and exclusion criteria

The PICOS (participants, intervention, comparator, outcome and study design) 23 approach was used to define the following eligibility criteria for study selection.

#### 2.1.1 Participants

Studies were included if participants lived in non‐Western countries (defined as countries that are not members of UN classification of Western European and Other States Group (WEOG)).^24^ The same classification method was used in a previous systematic review 25 We excluded studies that included children (aged <18).

#### 2.1.2 Interventions

We included studies which aimed to improve the participation of patients in the process of decision making. Studies were excluded if interventions: (a) only focused on information provision; (b) were about promoting self‐management of conditions; (c) were about patient participation in triadic decision making; and (d) aimed at promoting participation in clinical trials, patient safety measures or planning and development of health‐care programmes.

#### 2.1.3 Outcomes

Outcomes related to patient activation, patient or provider participatory behaviours during the decision‐making encounters were analysed. Patient activation is a broad concept with a definition of ‘an individual's knowledge, skill, and confidence for managing their health and health care’.^26^ In this systematic review, we only included studies which reported patient activation outcomes in relation to individuals’ skills and confidence in participating in health‐care decision making.

#### 2.1.4 Study designs

Randomized controlled trials (RCTs), controlled or uncontrolled before‐and‐after studies with pre‐ and post‐test data available and interrupted time series studies were included.

### 2.2 Search strategy and study selection

We systematically searched databases using keywords and Medical Subject Headings (MeSH) related to pre‐specified PICOS criteria. Some segments of our search strategy were adapted from other published systematic reviews with similar concepts.^4^, ^27^ The search strategy was originally developed in Medline via OvidSP (Appendix S2) and later modified to other databases. We initially limited our search to humans, adults and the English language, and later expanded the search to the non‐English language records. Returned records from database searches were combined, duplicates removed using Endnote X8 software, and remaining references imported to the Covidence tool^28^ for screening, data extraction and quality assessment purposes. Two reviewers conducted title and abstract screening and full‐text screening of eligible studies on Covidence. Disagreement on the selection of certain studies was resolved by consensus.

### 2.3 Data extraction and quality assessment

We extracted data using the Covidence online tool^28^ and an adaptation of the Cochrane Consumers and Communication Group data extraction template.^29^ We recorded country of origin, study design, participant numbers, intervention characteristics, theoretical framework, setting/conditions, outcome measures and detailed outcome results.

The quality of RCTs was assessed using the Cochrane risk of bias tool (sequence generation, allocation concealment, blinding, incomplete outcome data, selective outcome reporting and other sources of bias).^30^ The quality of non‐randomized studies was assessed using the modified Downs and Black's checklist,^31^ rating each study numerically against 27‐item questions, and the total score ranged from 0 to 28.

### 2.4 Data analysis

Due to the wide variation of study designs, intervention strategies and outcome measures used in the included studies, a narrative synthesis method was used. Narrative synthesis is a process of exploring study characteristics and their relationships within (and between) included studies in order to identify factors influencing the effectiveness and implementation of interventions.^32^ The process of narrative synthesis was partially guided by recommendations by Popay et al.^32^ We used textual description, grouping and tabulation methods for preliminary synthesis and exploration of patterns across studies.

## 3 RESULTS

### 3.1 Characteristics of included studies

**Figure 1:**
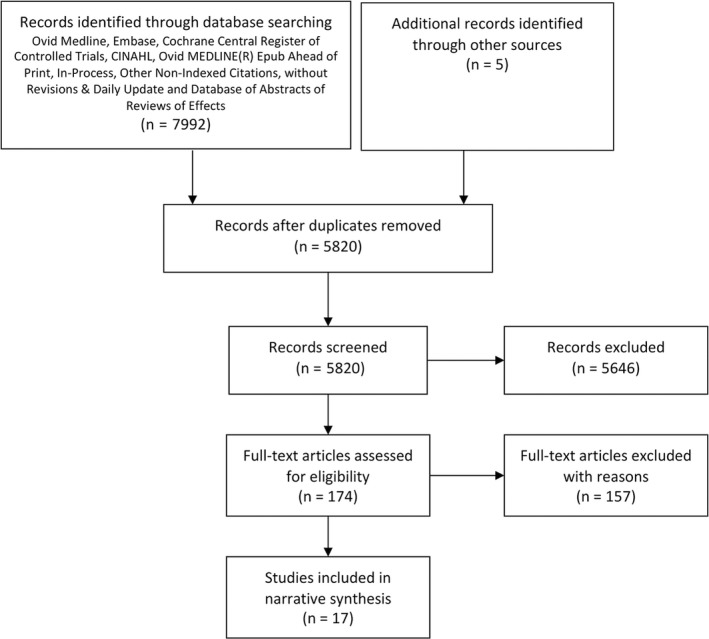
PRISMA flow diagram

A total of 7992 studies were identified through the initial search of publications in the English language. Two additional studies were added by recalling previously known studies and further three from citation searching. The search for non‐English language papers within the same databases identified 476 studies; however, none of these were eligible for inclusion. Seventeen studies (6 RCTs and 11 non‐RCTs) were included in the final stage of data extraction and quality assessment (Figure 1). The included studies were conducted in 10 countries, including Hong Kong and mainland China (n = 3), Japan (n = 3), South Korea (n = 2), Mexico (n = 2), Nicaragua (n = 1), Iran (n = 1), Indonesia (n = 2), Namibia (n = 1), Trinidad and Tobago (n = 1) and Honduras (n = 1). There were a variety of clinical conditions featured in these studies, including family planning (n = 5), general consultations (n = 3), breast cancer treatment (n = 2), dental consultations, carpal tunnel syndrome (CTS), primary open‐angle glaucoma (POAG), birth choice, mental health, HIV antiretroviral treatment and advanced care (see Table 1 and Table 2).

**Table 1:** Summary of intervention strategies used in included studies

Study	Theoretical framework	Target population	Intervention elements
Provider communication skills training			
Roter 1998^33^ (Trinidad & Tobago)	Interpersonal communication and counselling (IPC/C)	Ambulatory care doctors	Adapted from Continuing Education Program (CME) from the USA 8‐hour communication skills training for doctors Role‐play scenarios were incorporated into training Communication skills elements that were emphasized: information giving; emotional responsiveness and partnership building
Brown 2000^34^ (Honduras)	Interpersonal communication and counselling (IPC/C)	Ambulatory care doctors	Three half‐day interpersonal communication (IPC) training for providers Communication skills elements that were emphasized: socio‐emotional communication; problem‐solving skills; counselling Participatory training methods: participatory plenary sessions; presentations; role‐play; videotapes on non‐verbal aspects of communication; review of audio tapes of others’ and own patient consultations; job aid Cultural appropriateness of the training materials was consulted with local team.
Kim 2000^35^ (Indonesia)	Client‐centred care	family planning providers in rural areas	5‐day training workshop on client‐centred counselling One intervention group attended self‐assessment: 15‐20 min each week, focusing on one of the key communication areas, using a two‐page form Another intervention group attended weekly 30‐60 min of group peer review meetings to discuss issues related to self‐assessment The content of self‐assessment and peer review exercises is closely aligned with training content and providers were taught to how to do self‐assessment during the workshops
Kim 2002^36^ (Mexico)	Interpersonal communication and counselling (IPC/C)	Resident doctors working at rural clinics	Two‐day interpersonal communication skills training for resident doctors This training on communication skills had become an institutionalized part of standard resident training by Mexican Institute of Social Security/Solidarity (IMSS/S) Five‐day refresher course for resident doctors 5 months after the initial course Doctors in the intervention group received evaluations and feedback on their IPC/C communication skills from visiting supervisors who received 3‐day training on IPC/C and assessment of key communication skills Doctors in the intervention group were also instructed to audio tape two consultations a month and assess their performance using a job aid
Patient communication skills training			
Kim 2003^37^ (Indonesia)	Interpersonal communication and counselling (IPC/C)	Family planning clinics	Intervention was developed based on prior research, which suggested the need for individual coaching to tailor for health literacy needs and communication need for explicit permission to ask questions Patient educators coached patients in asking questions using a ‘smart patient’ leaflet Patients were coached on asking questions directly, asking for confirmation and writing down questions Providers of patients had previously participated in IPC/C training
Maclachlan 2016^38^ (Namibia)	Social cognitive theory of self‐efficacy (Bandura, 1977)	Hospitals with high HIV patient load	Three, 2‐hour patient education sessions on active participation Education curriculum was developed locally Curriculum content included the following: learning to speak to providers; using tools to help communication; overcoming barriers to communication
An 2017^39^ (South Korea)	Nil	Mental hospital	8‐session shared decision making (SDM) training programme for patients with schizophrenia was developed based on a previously developed programme elsewhere, and content was revised to suit the South Korean context Revised guidelines and textbooks on SDM had previously been distributed to community mental health facilities to promote effective patient‐provider communication Training activities included the following: explanation, role‐play, practising communication skills with their doctors, sharing experiences, giving presentations and giving feedback
Patient decision aids (± training)			
Lam 2013^40^ (Hong Kong, China)	International Patient Decision Aids Standards (IPDAS) Collaboration criteria	Government‐funded breast centres	A decision aid (DA) was developed based on previous research findings on breast cancer decision making amongst Chinese women and this decision aid followed (IPDAS) criteria DA was pilot‐tested and revised accordingly DA was for home‐use post‐consultation
Gong 2017^41^ (South Korea)	IPDAS criteria	Outpatient clinic at a tertiary referral setting	Patients were asked to view a 6‐minute video clip DA DA met most of the IPDAS criteria
Osaka 2017^42^ (Japan)	Ottawa Decision Support Framework (ODSF), IPDAS criteria, social comparison theory, social learning theory	Nil	A prototype DA with patient narratives was developed based on patient interviews and publicly existing breast surgery choice decision aids DA was intended for home‐use pre‐consultation
Kim 2005^43^ (Mexico)	Normative model of client‐provider communication for family planning decision making (unpublished)	Government health facilities	WHO developed family planning decision‐making tool (DMT) to be used during family planning consultations This DA was in a two‐sided flipchart format, with one side functioning as job aid for providers and other side acting as decision aid for patients Providers received DA and participated in 2.5‐day training on how to use the flipchart and some counselling skills
Kim 2007^44^ (Nicaragua)	Normative model of client‐provider communication for family planning decision making (unpublished)	Government health facilities	WHO developed family planning decision‐making tool to be used during family planning consultations This DA was in a two‐sided flipchart format, with one side functioning as job aid for providers and other side acting as decision aid for patients Providers received DA and participated in 3‐day training on how to use the flipchart and some counselling skills
Hu 2008^45^ (China)	nil	Public general dental hospital and individual clinics	A dental 3D multimedia system was developed based on a series of research on this topic The 3D multimedia system can display dental anatomy, explanations, animations, and advantages and disadvantages of relevant treatment options Dentists received training on how to use the tool, and they watched a videotape on communication with patients seeking prosthodontic treatment, covering areas of establishing rapport, showing empathy and making shared decisions
Farrokh‐Eslamlou 2014^46^ (Iran)	WHO DMT tool	Urban and rural public health facilities	WHO DMT was adapted to local context Providers participated in 2‐day workshop on how to use the flipchart
Shum 2017^47^ (Hong Kong, China)	IPDAS criteria	Ophthalmology outpatient clinic	A patient decision aid (PDA)was developed with consultation with specialists and was field‐tested with patients PDA met IPDAS criteria, and design was also guided by previous research on decision aid development for Chinese women Patients were given PDAs to read at‐home and were given 5‐min briefing on the content of the PDA
Torigoe 2016^48^ (Japan)	Ottawa Decision Support Framework (ODSF)	Obstetric institutions that permitted VBAC	Original Birth Choice Decision Aid Booklet and Ottawa Decision Support Guide (ODSG) were linguistically and culturally adapted Decision support programme, consisting of decisional needs assessment and decision support using decision aid booklet were provided
Question prompt material			
Shirai 2012^49^ (Japan)	Social cognitive theory of self‐efficacy (Bandura, 1977)	National Cancer Centre Hospital	Question prompt sheet was developed based on prior research Cancer patients were given question prompt sheet along with hospital introduction sheet upon being admitted to hospital

**Table 2:** Summary of included studies

Author year	Study design	Country	Relevant outcome measure/s	Number	Outcome	Change in decisional conflict/Preparedness	Patient participatory behaviours	Provider participatory behaviours	Quality Score
Provider communication skills training									
Roter 1998^33^	CBA	Trinidad and Tobago	Interaction analysis of audiotaped clinical encounters using RIAS, patient exit interviews, self‐administered questionnaire for health providers	18 doctors	Compared to untrained doctors, trained doctors experienced significant improvements in terms of using facilitators in their talk (change score: 3.12 vs −0.89, *P* = 0.015), using open questions (change score: 2.33 vs −0.35, *P* = 0.023) and being rated friendlier (change score: 0.75 vs −0.40, *P* = 0.007). No significant difference in change in medical information giving, medical and lifestyle counselling			↑	19
Brown 2000^34^	CBA	Honduras	Interaction analysis of audiotaped clinical encounters using RIAS, patient exit interviews, self‐administered questionnaire for health providers	49 health‐care providers, 220 patient consultations pre‐test, 218 post‐test	Compared to untrained doctors, trained doctors talked more (mean scores: 136.6 vs 94.4, *P* = 0.0001), used more positive talk (15.93 vs 7.99, *P* = 0.001), less negative talk (0.11 vs 0.59, *P* = 0.018), more emotional talk (15.7 vs 5.5, *P* = 0.021), and provided more medical counselling (17.3 vs 11.3, *P* = 0.026). Patients of trained doctors talked more (mean score: 113.8 vs 79.6, *P* = 0.011) and disclosed more medical information (54.7 vs 41.7, *P* = 0.002)		↑	↑	16
Kim 2000^36^	CBA	Indonesia	Interaction analysis of audiotaped clinical encounters using RIAS, provider interviews, patient exit interviews	201 providers from 170 clinics	Providers experienced significant increase in their frequency of facilitative communication after the training (from 15 to 30, *P* < 0.001). Clients of trained doctors experienced significant increase in their frequency of active communication (from 3.3 to 7.0, *P* < 0.001) and numbers of questions they asked (from 1.6 to 3.3, *P* < 0.001). Both the providers and clients in the self‐assessment and peer review groups experienced significant improvements in their facilitative communication and active communication, whilst the control group without reinforcement did not experience further improvement		↑	↑	13
Kim 2002^35^	CBA	Mexico	Interaction analysis of audiotaped clinical encounters using RIAS	60 doctors and 232 patients	Doctors in the intervention group experienced a 238% increase in their frequency of facilitative communication (from 13.6 to 45.9, *P* < 0.001), whilst the increase in the control group was 124% (14.6‐32.7, *P* < 0.001). After controlling for confounds, the increase was only significant in the intervention group but not in the control group. Frequency of patient participatory behaviours during consultations improved significantly from baseline to follow‐up with no significant differences between the intervention (from 2.4 to 12.7, *P* < 0.001) and control groups (from 2.6 to 13.0, *P* < 0.01)		↑	↑	18
Patient communication skills training/coaching									
Kim 2003^38^	Cluster RCT	Indonesia	Interaction analysis of audiotaped clinical encounters using RIAS; exit interviews	768 women, 384 in the intervention group, 384 in the control group	Compared to the control group, smart patient coaching patients asked significantly more questions (6.3 vs 4.9, *P* < 0.01) and expressed concerns and opinions (6.7C vs 5.4, *P* < 0.05). No difference in seeking clarification (1.8 vs 1.5)		↑		See Figure 2
Maclachlan 2016^37^	RCT	Namibia	Interaction analysis of audiotaped clinical encounters using RIAS	589 patients, 299 in the intervention group, 290 in the control group	Doctors of patients in the intervention group scored higher on facilitation and patient activation (adjusted difference in score 1.19, 95% CI 0.39‐1.99, *P* = 0.004) and gathered more information (adjusted difference in scores 2.96, 95% CI 1.42‐4.50, *P* = 0.000). Other doctor communication variables were also higher in the intervention group, however not statistically significant. Patients in the intervention group asked more questions (adjusted difference in score 0.48, 95% CI 0.11‐0.85, *P* = 0.012)		↑	↑	See Figure 2
An 2017^39^	CBA	South Korea	Administration of Self‐Esteem Scale and Problem‐Solving Inventory	29 in the intervention group, 31 in the control group	Compared to the control group, the intervention group achieved significantly more positive changes in self‐esteem (mean change ± SD: 4.06 ± 4.42 vs − 1.06 ± 3.66, *P* < 0.001) and problem‐solving (mean change ± SD: 17.31 ± 19.55 vs −0.54 ± 7.47, *P* < 0.001)	↑			21
Patient decision aid (±training)									
Lam 2013^42^	RCT	Hong Kong, China	Decision Conflict Scale; Videotape analysis using OPTION scale	138 women in the intervention group; 138 women in the control group	There was no significant difference in shared decision‐making OPTION scores of providers between the decision aid group and the control group (mean = 33.01, SD = 9.71 vs mean = 32.06, SD = 0.45). The decision aid group had significantly less decisional conflict at one‐week post‐intervention than the control group (mean = 15.8, SD = 15.5 vs mean = 19.9, SD = 16.3, *P* = 0.016). There was no difference in decision‐making difficulties between the intervention and control groups (17.5 vs 19.2, *P* = 0.064)	↑		↔	See Figure 2
Gong 2017^48^	RCT	South Korea	Decisional Conflict Scale	40 in the intervention group, 40 in the control group	There was no significant difference in decisional conflict scores between the intervention and control groups (22 vs 23, *P* = 0.76). The intervention group had significantly better knowledge than the treatment group (*P* = 0.04)	↔			See Figure 2
Osaka 2017^43^	RCT	Japan	Decisional Conflict Scale	210 women	Before the surgery and after the intervention, there was no significant difference in total decisional conflict scores between the decision aid, decision aid with narratives and control group (28.7 vs 29.8 vs 31.7). At 1 month post‐surgery, both the decision aid groups had significantly lower decisional conflict scores than the control group (26.5 vs 26.9 vs 32.1)	↑↔			See Figure 2
Kim 2005^40^	UCBA	Mexico	Interaction analysis of videotaped clinical encounters using Roter interaction analysis system (RIAS); Assessment of decision‐making process using adapted OPTION tool	13 providers; 35 consultations at baseline and 45 consultations post‐intervention	There were significant improvements (*P* < 0.001) in the numbers of sessions where minimum desired level of participatory behaviours by both the clients and providers were met after the intervention. For providers, significant changes in behaviours included the following: validating client preference (73.3% vs 0%), checking clients’ understandings (75.6% vs 0%) and discussing patient participation in decision (37.8% vs 0%). For clients, changes included acknowledging right to choose (40.0% vs 0%), deliberating preference (57.8% vs 0%) and seeking clarifications (68.9% vs 0%). Providers’ overall decision‐making score increased from 19 at baseline to 32 post‐intervention. Clients’ overall decision‐making score increased from 20 to 34 post‐intervention		↑	↑	14
Kim 2007^41^	UCBA	Nicaragua	Assessment of decision‐making process using adapted OPTION tool; Assessment of quality of consultations and key issues being discussed using client‐provider interaction (CPI) checklist	59 providers; 426 family planning clients	For new clients, providers’ overall decision‐making score increased significantly from 28.6 at baseline to 36.8 post‐intervention (*P* < 0.001). For continuing clients, providers’ overall score increased from 24.1 to 27.3 (*P* < 0.01). Both new and continuing clients experienced significant improvements in their own overall decision‐making scores (new clients: from 22.5 to 27.6, *P* < 0.001; continuing clients: from 18.1 to 19.9, *P* < 0.01). The intervention had greater impact on overall decision‐making performances of both providers and clients on sessions involving new clients compared to continuing clients		↑	↑	12
Hu 2008^47^	UCBA	China	Questionnaires assessing patient satisfaction, comprehension and perceptions	179 patients	Participants were more likely to rate themselves having participated in decision making after the intervention (OR 5.938, 95%, CI 2.741‐12.865) and at their second visit (OR 2.601, 95% CI 1.205‐5.614) compared to baseline		↑	↑	15
Farrokh‐Eslamlou 2014^44^	UCBA	Iran	Observation of consultations; exit interviews	448 clients at baseline and 547 clients post‐intervention	There were significant increases (*P* < 0.05) in the proportion of sessions where sufficient level of provider participatory behaviours were observed. These included significant increase in giving information about methods (from 58% to 80%), method efficacy (from 54% to 88%), how to use the chosen method (from 81% to 98%) and complication of the chosen method (from 67% to 94%). There were also significant increases in behaviours such as engaging clients to speak (from 75% to 93%) and answering all the client questions (from 89% to 99%)			↑	17
Shum 2017^45^	UCBA	Hong Kong, China	Decisional Conflict Scale	65 patients	There was significant reduction in decisional conflict after receiving the decision aid tool compared to baseline (mean decisional conflict score 34.3 ± 20.3 vs 48.9 ± 20.4, *P* < 0.01).	↑			16
Torigoe 2018^46^	UCBA	Japan	Modified Decisional Conflict Scale	33 women	There was significant reduction in decisional conflict after the decision support intervention compared to baseline (mean decisional conflict score: 2.18 ± 0.36 vs 2.54 ± 0.49, *P* < 0.001)	↑			17
Question prompt materials									
Shirai 2012^49^	RCT	Japan	Patient satisfaction with the consultation was assessed using five items adapted from a previous study. The number and contents of the questions were measured using interview method immediately after the consultation	32 cancer patients in the intervention and 31 in the control group	There was no difference between the Question Prompt Sheet (QPS) group and the Hospital Induction Sheet (HIS) group in terms of percentages of patients reporting had asked question (s) (63% vs 71%), numbers (both: median = 1, interquartile range = 2) and types of questions been asked and satisfaction with the consultation, such as satisfaction with asking questions (mean: 6.8 vs 7.8, *P* = 0.177). The QPS group rated the usefulness of the material in helping them asking questions significantly higher than the Hospital Induction Sheet (HIS) group (4.4 ± 3.6 vs 2.7 ± 2.8, *P* = 0.003)	↑	↔		See Figure 2

Abbreviations: CBA: controlled before‐and‐after studies; CI: confidence intervals; N: total number; OR: odds ratio; RCT: randomized controlled trial; RIAS: Roter interaction analysis system; RR: relative risk; SD: standard deviation; UCBA: uncontrolled before‐and‐after or time series studies; empty cells indicate that the outcome was not assessed; ↑ positive effect; ↔ no effect/difference.

**Figure 2:**
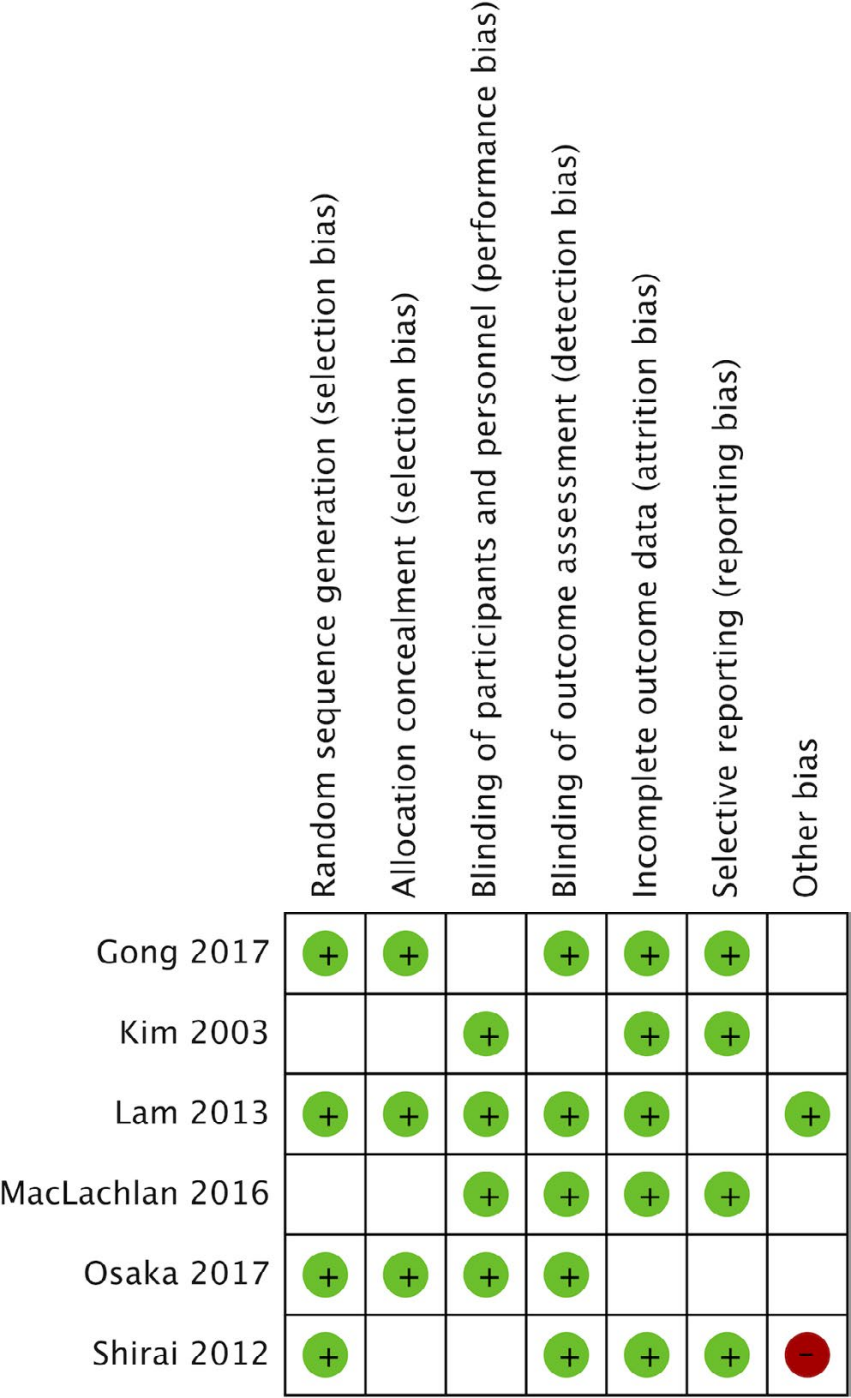
RCT studies rated against the Cochrane Risk of Bias tool 59. Green cells indicate low risk; red cells indicate high risk; blank cells indicate unclear risk

The methodological quality of the included RCTs varied across studies (Figure 2). Two (2/6) did not provide sufficient information on random sequence generation methods, and three (3/6) studies did not describe or have allocation concealment. Blinding of participants and personnel was lacking in two (2/6) studies, and in one study, outcome assessment was not reported in detail to permit a judgement. The Downs and Black quality scores for non‐randomized studies ranged from 12 to 21 (see Table 2).

### 3.2 Data synthesis

There were four types of intervention strategies in the included studies; provider communication skills training (n = 4), patient communication skills training (n = 3), question prompt material (n = 1) and patient decision aids (n = 9). Details of elements of each intervention strategies, theoretical background and development processes are summarized in Table 1. Based on our pre‐defined outcome inclusion criteria and emergent patterns in the extracted data, we categorized study outcomes into three groups: (a) change in decisional conflict or preparedness; (b) patient participatory behaviours; and (c) provider participatory behaviours (see Table 2).

#### 3.2.1 Provider communication skills training

Four studies used the conceptual framework of Interpersonal Communication and Counselling (IPC/C) 33, 34, 35 or client‐centred counselling^36^ for provider communication skills training, and all four were controlled before‐and‐after studies. Two studies^33^, ^34^ measured the effects of stand‐alone provider interpersonal communication skills training, whilst the other two^35^, ^36^ assessed the impact of self‐assessment, peer review and supervision on the maintenance of provider communication skills. They all used interaction analysis of audiotaped clinical encounters using the Roter interaction analysis system (RIAS). All four studies reported significant improvements in trained doctors’ facilitative talking behaviours, such as using open‐ended questions,^33^ facilitators (checking for understanding and asking for opinions),^33^, ^34^, ^35^ emotional talk^33^, ^34^ and partnership building^35^, ^36^ when compared with doctors in the control groups. Improvements in patient active communication, such as asking questions^36^ and providing medical information,^33^, ^34^ were also reported.

#### 3.2.2 Patient communication skills training

Three studies provided communication training for patients. One RCT from Namibia used a curriculum with three components: learning to speak to providers, using tools to help communication and overcoming barriers to communication.^37^ The second cluster‐randomized study from Indonesia provided individual coaching to patients on asking questions, requesting clarification and expressing concerns prior to their consultations.^38^ Finally, a controlled before‐and‐after study from South Korea developed a shared decision‐making training programme for people with schizophrenia.^39^

The Namibian training programme for patients resulted in the doctors of trained participants performing significantly better in facilitating (adjusted difference in score 1.19, *P* = 0.004) and gathering information (adjusted difference in scores 2.96, *P* = 0.000) than control group doctors. These trained patients also asked significantly more questions during consultations (adjusted difference in score 0.48, *P* = 0.012).^37^ The Indonesian study of individual coaching for patients also resulted in the coached patients asking significantly more questions than those in the control group (6.3 vs 4.9, *P* < 0.01).^38^ Similarly, the shared decision‐making training in South Korea found a significant positive change in self‐esteem in the intervention group compared to control (mean change ± SD: 4.06 ± 4.42 vs −1.06 ± 3.66, *P* < 0.001) which could be seen as empowerment in decision making.^39^

#### 3.2.3 Decision aids

Patient decision aids (PDAs) were utilized in nine of the 17 included studies and the PDAs were either paper‐based^40^, ^41^, ^42^, ^43^, ^44^, ^45^, ^46^ or computer‐delivered.^47^, ^48^ Five of these studies (5/9), all from East Asian countries, used the International Patient Decision Aids Standards (IPDAS) Collaboration checklist to guide the development of their decision aids.^42^, ^43^, ^45^, ^46^, ^48^ Of the nine included studies of PDAs, three were RCTs.^42^, ^43^, ^48^ We note that none of the RCTs^42^, ^43^, ^48^ included training as part of their intervention and that the studies which did were all of a weaker study design as uncontrolled before‐and‐after studies (n = 6).^40^, ^41^, ^44^, ^45^, ^46^, ^47^ Nevertheless, amongst these nine studies evaluating patient decision aids, there was a consistent improvement in the patient and provider participatory behaviours in those studies which included provider and/or patient training as part of the intervention.^40^, ^41^, ^44^, ^45^, ^46^, ^47^

The Hong Kong RCT found that the decision aid tool significantly reduced decisional conflict compared with a standard information booklet at one‐week post‐intervention (mean = 15.8, SD = 15.5 vs mean = 19.9, SD = 16.3, *P* = 0.016). However, there was no difference in providers' participatory behaviours, as analysed on consultation videotapes (mean = 33.01, SD = 9.71 vs mean = 32.06, SD = 0.45). The RCT from Japan found that decisional conflict was significantly reduced for both the decision aid groups one month after receiving them and after having the selected surgery (26.5 vs 26.9 vs 32.1), but not immediately after the intervention and before the surgery (28.7 vs 29.8 vs 31.7).^43^ Conversely, the RCT study from South Korea 48 which assessed a PDA for carpal tunnel syndrome did not find any difference in the decisional conflict between the PDA (video format) group and the regular information group (control) (22 vs 23, *P* = 0.76).

Amongst the remaining six uncontrolled before‐and‐after studies, three studies using the WHO family planning decision‐making tool (DMT) reported positive changes in provider and client participatory behaviours (see Table 2).^40^, ^41^, ^44^ The other two studies from China (PDA in POAG) and Japan (PDA in birth choices) reported a reduction in decisional conflict after patients were given PDAs with a short briefing^45^ and decisional support.^46^ The study from mainland China, using a 3D multimedia system able to display relevant dental anatomy as an aid for patient‐provider communication, reported that patients felt more involved in decision making, understood decisions and treatment planning.^47^

#### 3.2.4 Question prompt materials

One study (an RCT) in Japan compared the use of a Question Prompt Sheet (QPS) and standard Hospital Introduction Sheet (HIS) for advanced cancer patients.^49^ Participants who received the QPS were more likely to find the materials useful in helping them asking questions compared to the HIS group (4.4 ± 3.6 vs 2.7 ± 2.8, *P* = 0.003); however, there were no differences in asking questions (63% vs 71%) nor total questions asked (both median 1) between these two groups.^49^

## 4 DISCUSSION

This systematic review used a narrative synthesis method to summarize the effects of interventions for improving patient participation in health‐care decisions within non‐Western countries. Seventeen studies from 10 countries were included, covering a variety of health topics. These studies evaluated four main strategies, including patient or/and provider communication skills training, PDAs and question prompt material. We summarized the impact of these studies on three outcomes, namely patient decisional conflict, patient participatory behaviours and provider participatory behaviours.

Our findings show an evolution of patient participation research in non‐Western countries over the past two decades which parallels, but is less developed than in Western countries. For example, the earliest studies (Trinidad and Tobago 1998^33^; Honduras 2000^34^; Indonesia 2000^36^; Mexico 2002^35^) focused on training for health‐care providers in communication and counselling skills. These were funded by USAID, and two of these 33, 34 were part of a Quality Assurance Project replicating research from high‐income countries.^50^ Stand‐alone provider training based on IPC was found to be effective when characterized by two‐way communication, partnership, a caring environment and bridging the social distance.^33^, ^34^, ^50^ These findings were further validated by Kim and her colleagues, who incorporated ongoing supervision and self‐assessment of provider communication skills with the Interpersonal Communication and Counselling (IPC/C) training in Indonesia and Mexico and achieved long‐term maintenance of provider communication skills.^35^, ^36^ They suggested that incorporating specific supervision on provider IPC/C skills to the already existing functional provider performance supervision system could be a cost‐effective option in developing countries.^35^, ^36^

Communication skills training was evaluated amongst Indonesian patients of providers who had previously received client‐centred counselling skills training.^36^, ^38^ Patients received individual coaching which increased their participatory communication behaviours and confirmed the importance of explicit permission or endorsement from providers for patients to speak up and ask questions.^38^ This supports the notion that patient coaching could complement provider communication skills training in strengthening the partnership relationship during medical consultations.

A patient decision aid, the WHO family planning Decision‐Making Tool (DMT), began to be evaluated next in Mexico (2005),^40^ Nicaragua (2007)^41^ and Iran (2014).^44^ The DMT tool was developed by WHO in 2001 to promote client‐centred counselling and client active participation in decision making.^51^, ^52^ It has been translated into 20 languages and utilized by nearly 50 countries.^51^ These evaluative studies were developed as part of the USAID Information and Knowledge for Optimal Health (INFO) project, which had a mission of disseminating best practices in reproductive health care by facilitating knowledge sharing.^53^ The studies in Nicaragua 41 and Mexico^40^ showed that the use of the tool during family planning consultations improved provider participatory behaviours as well as patient participation in decision making.^40^, ^41^, ^44^, ^51^ Like the Indonesian study of patient coaching, these decision aid interventions included provider training on how to effectively use the tool and some briefings on counselling skills. The use of this tool was initiated by the health‐care providers during client encounters, which may have created an environment where clients felt safe and encouraged to play an active role. The more recent study from Iran also reinforced the positive effects of the DMT and provider training on patient participation in decision making.^44^

Our results highlight that the early studies in our review were international aid‐funded in low‐ and middle‐income settings, testing the transfer of Western country‐developed concepts such as patient‐centred counselling and shared decision making. The key effective components were as follows: (a) provider communication skills training in patient‐centred counselling; (b) ongoing supervision, peer review and self‐assessment of provider participatory communication and counselling skills; (c) targeted and tailored patient communication skills coaching or training; and (d) provision of a decision aid, to be used during the consultation by the provider.

By contrast, more recent studies included in this review (n = 9) were from high‐ and upper‐middle‐income East Asian countries (Japan, South Korea, Hong Kong and mainland China).^37^, ^42^, ^43^, ^45^, ^46^, ^48^, ^54^ Decision aids which met the IPDAS criteria were predominately tested in the studies from East Asia (5/8), especially since 2013.^42^, ^43^, ^45^, ^46^, ^48^ These studies mainly assessed the effect on decisional conflict; the three RCT studies^42^, ^43^, ^48^ within this group showed mixed and inconsistent results. However, as noted earlier, the non‐RCT PDA studies from East Asia which had provider^47^ or patient communication training^39^, ^45^, ^46^ components resulted in significant improvements to patient participation.

It is worth noting that a recent Special Issue on Shared Decision Making (SDM) published in conjunction with the International Shared Decision‐Making Conference in Lyon 2017 reported on the status of SDM implementation in over 20 countries.^55^ By contrast to the previous special issue in 2011,^56^ this issue included articles from several non‐Western countries and regions (Africa, Argentina, Brazil, Chile, China, Iran, Malaysia, Peru and Taiwan).^55^ Most of these countries reported a growing interest in patient participation but faced challenges in implementation. Therefore, at this time, there is a unique opportunity to expand and implement the evidence we have highlighted in this review. Ironically, the earliest work in non‐Western low‐income countries which showed considerable efficacy has not progressed and should be urgently revisited.

Our review was not able to explore specific cultural aspects of patient participation, but it did highlight the potential for this to be successfully achieved, particularly if provider training is incorporated. Learnings from the studies that used IPC should be particularly noted, due to the unique way that they emphasized reducing social distance, an aspect of culture not strongly featured in Western countries. Social distance can be a ‘virtual barrier’ between providers and patients created by the subjective feelings of alienation in class and status due to age, sex, race and social, educational, economic and cultural backgrounds.^50^ Therefore, such cultural and local aspects of patient‐provider communication in non‐Western should not be overlooked when designing interventions to promote patient active participation.

One of the limitations of this systematic review is that we focused our systematic review on participation in dyadic decision making amongst patients living in non‐Western countries. We acknowledge that family and significant others can play a significant role in the process of decision making in some patients from non‐Western cultural backgrounds.^57^ However, one recent study^58^ has suggested this may be less homogenous within cultures than previously thought. Another limitation is that we only included studies that were published in English and were identified from major databases in medical research. To draw more complete conclusions on the evidence from non‐Western countries, a comprehensive search of the literature in non‐Western country‐specific databases that collect local language studies might be needed in the future.

In conclusion, people in non‐Western countries can successfully be involved in their health‐care decisions, and this should not be overlooked as this is a core component of a people‐centred health‐care system as advocated by the Alma‐Ata Declaration and the WHO framework for IPCHS. Our study highlights the ability of communication skills training for patients and providers to increase patient participation and involvement in health‐care decisions. Such intervention strategies should be further developed and implemented as a priority in non‐Western countries regardless of their income status.

## CONFLICT OF INTEREST

No conflict of interest.

## Data Availability

Data for this systematic review were derived from published articles which are available in the public domain and may be subject to copyright. Relevant data supporting the conclusions of this systematic review are included within the article and supporting files.
