# Supplementary material for: Interventions to improve participation in health‐care decisions in non‐Western countries: A systematic review and narrative synthesis
Source: Health Expect. 2019 Jun 9;22(5):894–906. doi: 10.1111/hex.12933 (PMC6803415; doi:10.1111/hex.12933)
Supplement: Supplementary file 2 [file HEX-22-894-s002.docx]

**Ovid Medline Search Strategy**

Database(s): **Ovid MEDLINE(R)** 1946 to February Week 3 2018

Search Strategy:

| **#** | **Searches** | **Results** |
| --- | --- | --- |
| 1 | (non-Western or nonWestern).tw. | 1685 |
| 2 | exp Asia/ | 697107 |
| 3 | exp Europe, Eastern/ | 164585 |
| 4 | exp africa/ | 230038 |
| 5 | exp caribbean region/ or exp central america/ or exp "gulf of mexico"/ or exp latin america/ or exp south america/ | 178526 |
| 6 | exp african continental ancestry group/ or exp american native continental ancestry group/ or exp asian continental ancestry group/ or exp oceanic ancestry group/ | 160530 |
| 7 | exp Transcaucasia/ | 3935 |
| 8 | exp Mediterranean Islands/ not (Malta/ or Sicily/) | 1572 |
| 9 | exp Islands/ not (australia/ or great britain/ or iceland/ or new zealand/) | 271124 |
| 10 | Decision Making, Computer-Assisted/ | 2638 |
| 11 | Patient Education as Topic/ | 78790 |
| 12 | Decision Support Systems, Clinical/ | 6572 |
| 13 | exp Audiovisual Aids/ | 102032 |
| 14 | exp Decision Making/ | 173156 |
| 15 | Health communication/ | 1420 |
| 16 | ("shared decision making" or "shared decision-making").tw. | 3752 |
| 17 | (informed adj (decision* or choice*)).tw. | 6698 |
| 18 | decision support techniques/ | 17013 |
| 19 | ((patient$ or client$ or consumer$ or care?giver$ or carer$ or famil$) adj4 (pamphlet* or leaflet* or diary or diaries or sheet* or brochure$ or booklet* or "cue card*" or "issue card" or "option grid" or prompt* or checklist* or audio$ or recording$ or video$ or tape$ or taping or summar*)).tw. | 33882 |
| 20 | ((patient$ or client$ or consumer$ or care?giver$ or carer$ or famil$) adj4 (decision adj1 (aid* or tool* or board* or support*))).tw. | 1345 |
| 21 | ((patient$ or client$ or consumer$ or care?giver$ or carer$ or famil$) adj5 "motivational interviewing").tw. | 307 |
| 22 | patient-centered care/ or ((patient* or famil*) adj2 (centered or centred)).tw. | 25317 |
| 23 | ((patient$ or client$ or consumer$ or care?giver$ or carer$ or famil$) adj3 (educat$ or inform$ or train$ or counsel$ or workshop or coach* or encourage* or facilitat* or involv* or engage* or participat* or collaborat*)).tw. | 209812 |
| 24 | 10 or 11 or 12 or 13 or 14 or 15 or 16 or 17 or 18 or 19 or 20 or 21 or 22 or 23 | 604902 |
| 25 | Patient Participation/ | 21980 |
| 26 | patient satisfaction/ | 71786 |
| 27 | Consumer Participation/ | 15563 |
| 28 | ((patient$ or client$ or consumer$ or care?giver$ or carer$ or famil$) adj3 (participat$ or shar$ or joint or empower$ or involve$ or activat$ or engage* or communicat$ or interact$ or attitude$ or ask$ or question$ or preference$ or choice$ or decision$)).tw. | 214545 |
| 29 | (decision* adj1 (regret or satisfaction or conflict or confidence)).tw. | 922 |
| 30 | exp Professional-Patient Relations/ | 132693 |
| 31 | exp Decision Making/ | 173156 |
| 32 | 25 or 26 or 27 or 28 or 29 or 30 or 31 | 558594 |
| 33 | Interrupted time series.ti,ab. | 1640 |
| 34 | Controlled trial.ti,ab. | 85868 |
| 35 | parallel group trial.ti,ab. | 1205 |
| 36 | Program Evaluation/ | 55567 |
| 37 | (before adj2 after adj2 (stud* or trial* or design*)).ti,ab. | 8648 |
| 38 | Random Allocation/ | 93229 |
| 39 | Single-Blind Method/ | 24594 |
| 40 | Double-Blind Method/ | 144044 |
| 41 | control groups/ | 1572 |
| 42 | (time adj2 series).ti,ab. | 19232 |
| 43 | (pre-test or pretest or "pre test" or pre-intervention or preintervention or "pre intervention" or post-intervention or postintervention or "post intervention" or posttest or post-test or "post intervention").ti,ab. | 33014 |
| 44 | (pre post or prepost).tw. | 6362 |
| 45 | (quasi-experiment$ or quasi-random).ti,ab. | 8078 |
| 46 | (non randomi?ed or nonrandomi?ed or non-randomi?ed).tw. | 17876 |
| 47 | follow-up studies/ | 583529 |
| 48 | randomized controlled trial.pt. | 453101 |
| 49 | controlled clinical trial.pt. | 92131 |
| 50 | clinical trials as topic.sh. | 182496 |
| 51 | trial*.tw. | 742297 |
| 52 | randomi?ed.tw. | 451095 |
| 53 | Comparative Study/ | 1789116 |
| 54 | Evaluation Studies/ | 231970 |
| 55 | 33 or 34 or 35 or 36 or 37 or 38 or 39 or 40 or 41 or 42 or 43 or 44 or 45 or 46 or 47 or 48 or 49 or 50 or 51 or 52 or 53 or 54 | 3488015 |
| 56 | exp animals/ not humans/ | 4424434 |
| 57 | 55 not 56 | 2959162 |
| 58 | 1 or 2 or 3 or 4 or 5 or 6 or 7 or 8 or 9 | 1396928 |
| 59 | 24 and 32 and 57 and 58 | 4400 |
| 60 | limit 59 to (english language and "all adult (19 plus years)") | 2796 |
